# Rational Design and One-Step Immobilization of Chitosanase for Specific and Recyclable Chitobiose Production

**DOI:** 10.3390/foods14244248

**Published:** 2025-12-10

**Authors:** Dandan Tang, Jie Zhang, Na Li, Rui Long, Xinyu Wang, Xiaowen Wang, Wei Liu

**Affiliations:** 1 College of Food Science and Light Industry, Nanjing Tech University, Nanjing 211816, China; tangdandan@njtech.edu.cn (D.T.); 202361119014@njtech.edu.cn (J.Z.); 202161118046@njtech.edu.cn (N.L.); 202361119020@njtech.edu.cn (R.L.); 202421058007@njtech.edu.cn (X.W.); wxw24@njtech.edu.cn (X.W.); 2 School of Pharmaceutical Sciences, Nanjing Tech University, Nanjing 211816, China

**Keywords:** chitosanase, chitobiose, enzyme immobilization, elastin-like polypeptide, biomimetic silica

## Abstract

Chitosan oligosaccharides (COSs) with defined degrees of polymerization (DP) exhibit distinct bioactivities with promising applications in food, pharmaceutical, and agricultural industries. However, the specific and sustainable production of COSs remains challenging due to the broad product distribution of wild-type chitosanases and the difficulties in enzyme recovery and reuse. In this study, we employed rational design to engineer a GH46 chitosanase (CsnB) from *Bacillus* sp. BY01 for chitobiose production. Through homology modeling and molecular docking analysis, 15 mutants were designed by targeting key residues structurally critical for substrate stabilization, product release, and active-site geometry in the substrate-binding subsites. The D78Y mutant exhibited exclusive specificity for chitobiose, demonstrating a specific activity of 102.4 U/mg and yielding chitobiose with a purity exceeding 98%, thereby surpassing the previously reported enzymes for chitobiose production. To address the challenges of enzyme stability, purification costs, and product separation, we developed a ReELP system by integrating elastin-like polypeptides (ELPs) with a ReverseCatcher/ReverseTag peptide pair. This system enabled one-step purification and co-immobilization of CsnB-D78Y directly from cell lysate onto biomimetic silica nanoparticles, achieving 96.8% immobilization efficiency and 90.7% activity recovery. The immobilized enzyme exhibited enhanced thermal and pH stability, retaining approximately 50% activity after 12 h at 40 °C compared to only 5.7% for the free enzyme. In reusability assays, the immobilized CsnB-D78Y maintained efficient chitobiose production over 5 consecutive cycles. This work provides a green and cost-effective strategy for the specific and sustainable production of chitobiose, offering new insights into enzyme engineering and immobilization for industrial COS production.

## 1. Introduction

Chitosan is a linear polysaccharide obtained through strong alkaline treatment or partial deacetylation of chitin. It consists of alternating N-acetyl glucosamine (GlcNAc) and glucosamine (GlcN) residues linked by β-1,4-glycosidic bonds [1]. Upon depolymerization, it generates chitosan oligosaccharides (COSs). Due to their potential for inhibiting microbial growth [2], antioxidant activity [3], anticancer properties [4], anti-inflammatory effects [5], regulation of blood pressure and lipids, and plant defense, COSs have become a multifunctional carbohydrate of significant interest in the food, agricultural, and pharmaceutical industries. However, COSs produced by chemical methods are not environmentally friendly [6], whereas enzymatic production offers a more sustainable and mild approach [7].

Chitosanase specifically hydrolyzes the β-1,4-glycosidic bonds in chitosan to produce COSs with varying degrees of polymerization (DP) [8]. These enzymes are categorized into seven glycoside hydrolase (GH) families: GH 2, GH 5, GH 7, GH 8, GH 46, GH 75, and GH 80, with GH46 being the most extensively studied [9]. The primary enzymatic products are usually chitobiose and chitotriose [10,11]. Research suggests that the DP of COSs is a key factor influencing their physicochemical properties and bioactivities [12]. Chitobiose has been demonstrated to exhibit superior bioavailability compared to other high DP COSs [12,13]. Nevertheless, the acquisition of pure chitobiose remains challenging due to the extensive structural and chemical similarities among various chitin oligosaccharide species, which complicate separation and purification processes. Among the reported wild-type chitosanases, only the novel chitosanase Csn-PD from *Paenibacillus dendritiformis* exclusively produces pure (GlcN)_2_ [14], while most other endo-chitosanases generate both chitobiose and chitotriose. To obtain COS with specific DP, apart from discovering new enzymes that produce single products, protein engineering has been shown to be an effective approach for modifying chitosanase activity.

Additionally, the industrial application of chitosanases is hindered by challenges such as limited stability, high costs, harsh operational conditions, and difficulties in recovering free enzymes from viscous chitosan hydrolysates. Enzyme immobilization offers a promising solution by reducing enzyme consumption, simplifying downstream processes, and enhancing operational stability [15,16]. The simplicity and cost-effectiveness of immobilization techniques are critical for their industrial application. Silica-based materials have garnered significant interest due to their simple and eco-friendly preparation methods, with potential applications in co-immobilization, biosensors, and biomedicine [17,18]. One notable challenge in enzyme immobilization is the high cost of enzyme purification. This makes the search for an integrated solution that can simultaneously achieve efficient purification and immobilization a crucial pursuit in the field.

Peptide-based purification techniques provide fresh perspectives in this regard. Research has shown that mineralizing peptides can be genetically modified to quickly create functional silica materials by fusing with target proteins [19]. One such peptide, elastin-like polypeptide (ELP), was created to resemble elastin and is extremely sensitive to temperature variations. The repeating pentapeptide units that make up its structure are Val-Pro-Gly-Xaa-Gly (VPGXG), where Xaa can be any amino acid except proline [20]. The inverse temperature transition (ITT), a quick and reversible phase transition from a soluble to an insoluble state at a particular temperature, is one of the most significant characteristics of ELPs. ELPs conduct a reversible self-assembly process to form aggregates above this temperature, but below it they dissolve in aqueous solution. By altering the amino acid content and sequence length of ELPs, the ITT can be controlled [21]. In addition to its reversible phase transition properties, cationic ELPs [KV8F-40] (comprising 40 repeats of the pentapeptide motif) have been shown to rapidly prepare biomimetic silica nanoparticles, with mineralization activity positively correlated with the content of basic amino acids [22]. These characteristics are simpler and less expensive than traditional purification methods that rely on the fusion of various short peptide tags, such as His and GST tags. These characteristics make ELPs excellent scaffolding for developing temperature-responsive systems that can be activated by heating.

Additionally, peptide pairs have emerged as a potent immobilization method in enzyme engineering, especially for the covalent immobilization of numerous enzymes, due to their ability to conjugate highly specific enzymes both in vitro and in vivo via isopeptide bonds. These peptides allow site-specific conjugation without purification and are more environmentally friendly than chemical cross-linking. SpyTag/SpyCatcher [23] and SnoopTag/SnoopCatcher [24] are examples of common peptide pairs. Based on an ester bond [25], which can be hydrolyzed under specific conditions, our team created the unique peptide pair ReverseCatcher/ReverseTag, which effectively cyclizes proteins, increases their thermal stability [26]. This system, combined with ELPs, can simultaneously achieve the integration of efficient purification and immobilization.

In this study, we selected the wild-type chitosanase CsnB from *Bacillus* sp. BY01, a member of the GH46 family, to examine its enzymatic properties and product distribution. By rational design, we identified the active site of CsnB and performed site-directed mutagenesis to optimize product distribution, ultimately obtaining chitobiose as the main product. To enhance the stability of the mutated chitosanase and address the challenges of enzyme and product separation, we combined ELP with the ReverseCatcher/ReverseTag, serving as both a purification tag and immobilization carrier. This allowed us to explore a one-step process for simultaneous purification and co-immobilization of chitosanase for chitobiose production from chitosan, achieving improved stability and recyclability. The findings provide a foundation for future research on the green and sustainable conversion of chitosan to COSs with defined DP.

## 2. Materials and Methods

### 2.1. Materials and Medium

Luria–Bertani (LB) medium containing 10 g/L tryptone, 5 g/L yeast extract, and 10 g/L NaCl was used for *Escherichia coli* cultivation. The solid LB medium containing additional 2% agar powder was used for transformants screening. Chitosan (the degree of deacetylation (DDA) >75%) was purchased from Samarium Chemical Technology Co., Ltd. (Shanghai, China). Chitobiose (GlcN)_2_, chitotriose (GlcN)_3_, chitotetraose (GlcN)_4_, chitopentaose (GlcN)_5_, and chitohexaose (GlcN)_6_, were all purchased from Aladdin (Shanghai, China). Phanta Max Super-Fidelity DNA polymerase used for gene amplification was purchased from Vazyme (Nanjing, China). The remaining unlisted chemical reagents were from Sigma-Aldrich (Shanghai, China).

### 2.2. Plasmids Construction

The chitosanase (CsnB) from *Bacillus* sp. BY01 (GenBanK: MN531545) and *elp* gene (derived from K5V4F, GenBank: MN13629) were chemically synthesized by General Biosystems Co., Ltd. (Anhui, China) and cloned into the pET-22b and pET-28a vectors, respectively. The recombinant expression vector pET-22b-CsnB was used as a template to produce mutants by site-directed mutagenesis. The primers used for all mutants are shown in Table S1. ReverseCatcher was amplified from the pET-22b-ReverseCatcher-MPB plasmid [27] and fused to the N-terminus of ELP using a (GGGGS)_3_ flexible linker to construct plasmid pET-28a-ReverseCatcher-ELP. The construction of pET-22b-ReverseTag-CsnB-D78Y involved fusing the ReverseTag and (GGGGS)_3_ flexible linker to the C-terminus of CsnB-D78Y.

### 2.3. Protein Expression, and Purification

*E. coli* BL21 (DE3) were purchased from Vazyme (Nanjing, China) for proteins expression. The recombinants were cultured in 50 mL LB medium containing ampicillin (50 μg/mL) at 37 °C with shaking at 200 rpm. When the OD_600_ reached 0.5–0.8, isopropyl β-D-1-thiogalactopyranoside (IPTG, 0.25 mM) was added to induce protein expression. The cells were further incubated for 16–20 h at 20 °C and then collected by centrifugation at 8000 g for 5 min. A volume of 5 mL of Tris-HCl (50 mM, pH 7.0) was utilized to resuspend the cell pellet. Cell lysis was accomplished exclusively by sonication on ice using a cycle of 3 s of operation followed by a 5 s interval, for a total duration of 10 to 15 min. As for pET-22b-ReverseTag-CsnB-D78A, pET-22b-CsnB and the derived mutants, the proteins purification procedures were similar to those previously reported [27]. The purification of ReverseCatcher-ELP (RCE) was performed using the inverse transition cycling (ITC) method. First, 5 M NaCl solution was mixed with the cell lysate to achieve a final NaCl concentration of 2.5 M. The mixture was incubated in a 40 °C water bath for 3 h to induce phase separation and precipitation of ReverseCatcher-ELP through its thermoresponsive phase transition. The mixture was centrifuged at room temperature (12,000× *g*, 10 min) to collect the aggregated ReverseCatcher-ELP precipitate. The precipitate was resuspended in chilled 50 mM PBS (pH 7.0), then stored at 4 °C for 1 h. Subsequently, the suspension was centrifuged again at 4 °C and 12,000× *g* for 20 min to remove contaminating protein precipitates. The resulting supernatant contained the purified RCE protein.

### 2.4. Chitosanase Activity Assay

The chitosanase activity was evaluated by the 3,5-dinitrosalicylic acid (DNS) method [28]. A 50 μL portion of properly diluted enzyme solution was incubated with 350 μL of 0.5% (*w*/*v*) chitosan at pH 6.0 and 40 °C for 10 min. The reaction was terminated by adding 600 μL DNS reagent, followed by boiling in a water bath for 10 min, and then centrifuged at 12,000× *g* for 5 min. The absorbance of the supernatant after cooling was measured at 540 nm (A540) using a SpectraMax M3 microplate reader (Molecular Devices, San Jose, CA, USA). One unit of chitosanase activity (U/mL) was defined as the amount of enzyme required to produce 1 μmol reducing sugar per minute under the above conditions.

### 2.5. Homology Model and Molecular Docking

The three-dimensional structure of chitosanase CsnB (GenBank: MN531545) was modeled using the SWISS-MODEL web server (https://swissmodel.expasy.org/ (accessed on 3 April 2024)) with the ProteinData Bank (PDB) template 2D05 (sequence similarity: 99.57%). The substrate employed was chitohexaose (GlcN)_6_ (PubChem CID: 100978292). Molecular docking was performed using AutoDock Vina 1.1.2 with a grid box of dimensions 60 × 50 × 60 Å and spacing of 0.375 Å. The complex with the lowest binding energy was selected for analysis of active-site critical residues. Structure-based site-directed mutagenesis targeting the active site was conducted using PyMOL Version 2.5.0a0. (https://pymol.org/ (accessed on 15 April 2024)) to unveil substrate-binding influences on enzymatic activity and catalytic mechanisms.

### 2.6. Preparation and Characterization of Self-Assembled RCE Silica Particles

The methodology for synthesizing ELP-mediated silica particles was adapted from Lin et al. [29]. Orthosilicic acid stock was prepared by diluting 1 M tetramethyl orthosilicate (TMOS) with HCl to achieve a final concentration of 1 mM. This solution was mixed with diluted RCE in a 9:1 (*v*/*v*) ratio under neutral pH conditions at room temperature for 5 min. A control sample was prepared by substituting ELP with PBS buffer. The mixture was centrifuged at 12,000× *g* for 2 min at 4 °C, with the supernatant retained for quantifying unencapsulated RCE. The precipitate was washed with deionized water to remove excess silica and unbound RCE, then resuspended in PBS to restore the original volume.

To evaluate RCE’s influence on silica precipitation, fresh RCE solutions (0, 50, 100, 150, and 200 μM) underwent mineralization. Experiments were conducted in triplicate, and RCE immobilization efficiency was calculated via the formula:

(1) $$\mathrm{Immobilization}\;\mathrm{efficiency}\;(\%)\;=\;\frac{m_{i}-m_{s}}{m_{i}}\times 100$$

where m_i_ is the initial amount of RCE, and m_s_ is the amount of unimmobilized RCE in the supernatant after centrifugation.

To evaluate leakage profiles, RCE@SiO_2_ nanoparticles (NPs) dispersed in PBS were stored at 4 °C and 25 °C. At pre-determined time points (0, 12, 24, 36, and 48 h), the samples were centrifuged at 12,000× *g* for 2 min at 4 °C. Supernatants were collected for quantification of released protein and calculation of leaching rates. The post-centrifugation RCE@SiO_2_ NPs were further characterized using scanning electron microscopy (SEM) as previously described [30].

### 2.7. Effect of Temperature/pH on the Activity of Free and Immobilized CsnB-D78Y

To investigate the impact of temperature on the activity of free and immobilized CsnB-D78Y, the enzyme solutions were mixed uniformly with 0.5% (*w*/*v*) chitosan at pH 6.0, followed by incubation in a water bath at 20 °C, 30 °C, 40 °C, 50 °C, 60 °C, and 70 °C for 15 min, respectively. Triplicate experiments were conducted for each condition. The relative enzyme activities of free and immobilized enzyme at different temperature were calculated by comparing them to the maximum activity of free enzyme (set as 100% at its optimal temperature).

To investigate the impact of pH on the activity of free and immobilized CsnB-D78Y, the enzyme solutions were mixed uniformly with 0.5% (*w*/*v*) chitosan and incubated at 40 °C with a pH range of 4–9, respectively. Triplicate experiments were conducted for each condition. The relative enzyme activities of free and immobilized enzyme at different pH were calculated by comparing them to the maximum activity of free enzyme (set as 100% at its optimal pH).

To access the stability of free and immobilized CsnB-D78Y, the enzyme solutions were incubated in PBS buffer (pH 7, 50 mM) at 40 °C for 2 h, 4 h, 6 h, 12 h, and the samples were collected to determine the remaining enzyme activity. Triplicate experiments were conducted for each condition. The samples stored at 40 °C for 0 h were chosen as the control, and the enzyme activity was designated as 100%.

### 2.8. One-Step Purification and Immobilization of CsnB-D78Y from Cell Lysate

Since ReverseCatcher and ReverseTag can quickly form stable ester bonds at room temperature, the purification and immobilization of ChiRT (CsnB-D78Y-ReverseTag) were modified based on Cai’s study [30]. The RCE@SiO_2_ NPs were incubated with crude ChiRT at room temperature in PBS buffer (80 mM, pH 6.0) containing 20% glycerol, and 100 μM CaCl_2_ for 1 h. After incubation, the mixture was centrifuged to collect the silica NPs containing immobilized ChiRT (ChiRT-RCE@SiO_2_ NPs), and the supernatant was stored to quantify the immobilized effect by SDS-PAGE. The activity of the unimmobilized and immobilized enzyme in PBS buffer were determined using the DNS method. The activity recovery and immobilization efficiency were calculated as previously reported [31].

### 2.9. The Reusability of Immobilized CsnB-D78Y for Chitobiose Production

The reusability of immobilized CsnB-D78Y was evaluated in a 5-cycles repeated batch experiment (40 °C and pH 6.0). After each cycle, the ChiRT-RCE@SiO_2_ NPs containing the immobilized CsnB-D78Y was harvested and resuspended in the fresh 1 g/L colloidal chitosan solution to start a new cycle. The activity of the first cycle was defined as 100%. All experiments were carried out in triplicate.

### 2.10. Hydrolytic Products Analysis

The hydrolytic products of chitosan were analyzed by thin layer chromatography (TLC) or high-performance liquid chromatography (HPLC) methods. As for TLC analysis, 0.5% (*w*/*v*) chitosan were incubated with the purified enzymes at 40 °C. After different reaction times, the samples were picked out and boiled. The standard (GlcN)_2_-(GlcN)_6_ was used as control, and 15 μL of the samples were spotted onto a silica gel sheet and put into the developing solvent. The developing solvent was a mixture of ammonia, water, and isopropanol in volume ratios of 3:27:70. Then 0.5% ninhydrin ethanol solution was sprayed onto the TLC plate after drying and the plate was heated at 80 °C for 30 min for final visualization.

HPLC analysis was performed using a Shodex Asahipak NH2P-504E chromatography column (Shanghai, China) (4.6 mm × 250 mm, 5 μm particle size). The mobile phase consisted of 75% acetonitrile, with an evaporative light scattering detector (ELSD) for detection. The flow rate was maintained at 1.0 mL/min, and the column temperature was set to 30 °C. Samples were injected in 10 μL portions.

## 3. Results and Discussion

### 3.1. Structural Analysis of CsnB via Homology Modeling and Molecular Docking

**Figure 1:**
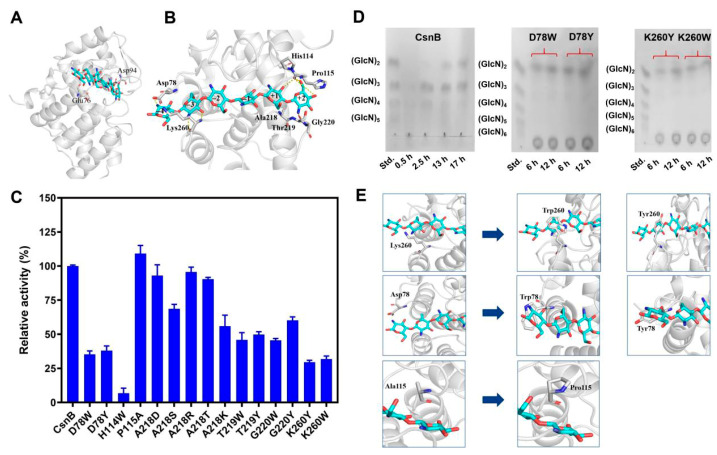
(**A**) The overall structure of the CsnB-substrate chitohexaose complex. Chitosanase is rendered in a ribbon representation, with catalytic residues Glu76 and Asp94 colored light gray. The substrate chitohexaose is displayed as cyan-colored rod models. (**B**) The hydrogen-bonding interactions within the catalytic cleft of the CsnB-substrate complex. Chitosanase was depicted in a ribbon representation with light gray coloring, while the substrate chitohexaose was shown in cyan-colored rod displays. Each mutated residue position was highlighted in rod format with light gray shading. (**C**) Comparison of enzyme activities of the wild-type CsnB and the mutants. (**D**) Hydrolysis products analysis of CsnB and the mutants CsnB-D78W, CsnB-D78Y, CsnB-K260Y, CsnB-K260W. (**E**) Changes in protein structure of mutants CsnB-P115A, CsnB-D78W, CsnB-D78Y, CsnB-K260W and CsnB-K260Y. Residues Pro115, Asp78, Lys260 and their mutants P115A, D78W, D78Y, K260W, and K260Y were displayed as light gray rod models.

Protein BLAST analysis (https://blast.ncbi.nlm.nih.gov/Blast.cgi (accessed on 15 March 2024)) of the CsnB sequence from *Bacillus* sp. BY01 revealed significant sequence homology with the GH46 chitosanase PDB entry 2D05 (resolution: 1.6 Å), sharing identical secondary structural organization. This structural similarity justified the selection of 2D05 as the homology modeling template. The predicted CsnB structure (Figure 1A), generated via SWISS-MODEL, exhibited a dual-domain architecture with an upper and lower domain, forming a prominent catalytic cleft at the active site, a defining feature of GH46 chitosanases. The conserved catalytic residues Glu76 and Asp94 served as general acid/base pairs to mediate the enzymatic reaction through proton shuttle mechanisms.

The complex structure of CsnB bound to (GlcN)_6_ revealed the substrate localized in the catalytic cleft between its two domains, comprising six glucosamine residues sequentially labeled from subsites (−4) to (+2) (Figure 1B). Computational docking analysis identified the proposed cleavage position between subsites (−2) and (−1). Notably, terminal residues of (GlcN)_6_ established hydrogen bonds with the (−4)–(−3) and (+1)–(+2) subsite residues, prompting the selection of residues Lys260 and Asp78 and adjacent residues (Ala218/Thr219/Gly220) as structural targets. Mutagenesis of these residues at the catalytic cleft extremities aims to alter the enzyme’s conformational constraints, thereby modulating its product specificity toward desired hydrolysis outcomes.

### 3.2. Rational Design and Functional Characterization of CsnB Mutants

To gain insights into the structural determinants of product specificity, 15 CsnB mutants were rationally designed by targeting residues located at or near the catalytic cleft, particularly those forming the (−3), (−2), (−1), and (+1) subsites, which were predicted to interact with glucosamine units during catalysis. Residues Asp78, His114, Pro115, Ala218, Thr219, Gly220, and Lys260 were selected for substitution due to their potential roles in substrate stabilization, product release, and shaping of the active-site geometry.

The specific activities of wild-type and mutant enzymes were determined using colloidal chitosan as substrate under standard conditions. As shown in Figure 1C, mutations exerted varying effects on catalytic performance. The wild-type enzyme exhibited robust activity (269.5 U/mg), set as 100% for relative comparisons. Among the variants, H114W retained only 6.85% of wild-type activity, confirming the essential role of His114 in stabilizing substrate binding through hydrogen bonds with the 2-amino and 3-hydroxy groups of glucosamine. Loss of this interaction led to a near-complete disruption of catalysis [32]. In contrast, P115A displayed an enhanced specific activity (109.1% of wild-type). Pro115, originally forming part of a rigid loop behind the active site, limits the flexibility required for efficient substrate accommodation. Its replacement by alanine likely increased loop mobility, allowing closer enzyme-substrate interactions and faster turnover.

Substitution of Thr219, and Gly220 with bulky aromatic residues (Trp or Tyr) led to a pronounced loss of activity. Structural modeling suggested that these residues might introduce steric hindrance that blocked the entry or proper positioning of longer oligomers, leading to restricted catalysis. Similarly, substitutions at Asp78 (D78W, D78Y) and Lys260 (K260W, K260Y) lowered activity to approximately 29.6% to 38.0% of wild-type levels. These residues are positioned near the (−4)/(−3) subsites, and their mutations likely disturbed substrate orientation and reduced binding affinity. Mutants at Ala218 with residues of different side-chain properties (R, K, S, T, D) retained moderate activity, suggesting that while this site contributes to substrate stabilization, its chemical environment can tolerate certain variations [8].

### 3.3. Hydrolysis Product Profiles by CsnB Mutants

The hydrolysis products generated by wild-type and the mutants were analyzed by TLC after incubation with colloidal chitosan (Figure 1D and Figure S1). For the wild-type enzyme, CsnB, early hydrolysis primarily yielded (GlcN)_3_ and (GlcN)_4_, which shifted to a mixture of (GlcN)_2_ and (GlcN)_3_ after prolonged reaction. Notably, no monomeric glucosamine was detected, confirming the enzyme’s endo-type cleavage mode. Specifically, the products of D78W, D78Y, K260W, and K260Y were primarily chitobiose. Thus, we can conclude that altering the (−4)/(−3) subsite structure successfully altered the enzyme’s product profile, resulting in the selective release of DP2. Notably, although CsnB-D78Y exhibited lower enzyme activity than the wild-type enzyme, it demonstrated a specific activity of 102.4 U/mg, surpassing that of the previously reported chitobiose-producing enzyme Csn-PD (76.4 U/mg) from *Paenibacillus dendritiformis* [14] as well as the MH-K1 mutant from *Bacillus circulans* [33]. These results suggest that CsnB-D78Y is a promising candidate for efficient (GlcN)_2_ production. Other mutants primarily generated (GlcN)_2_ and (GlcN)_3_ as hydrolysis products (Figure S1). In contrast, the P115A mutant exhibited enhanced hydrolytic efficiency, yielding a higher proportion of chitotriose (~60% of total products) compared to the wild-type enzyme (~30%). This improvement suggests that increased flexibility in the substrate-binding loop promotes the cleavage of larger oligosaccharides. Consequently, CsnB-P115A represents a promising candidate for targeted engineering to optimize chitotriose production.

To rationalize the observed changes, homology modeling and structural simulations were performed for P115, D78 and K260 (Figure 1E). Structural analysis of the P115A variant highlighted an increased openness and flexibility in the loop regions flanking the catalytic cleft, which correlates with its enhanced catalytic efficiency and greater production of high-molecular-weight hydrolysis products. Substitution of residues D78 and K260 with Trp/Y (D78W/Y and K260W/Y) introduced aromatic rings that stabilized the substrate’s positioning through hydrophobic interactions at corresponding subsites. By counteracting substrate slippage during non-processive hydrolysis, these interactions likely produced preferential cleavage at shorter glycosidic linkages. However, the bulkier side chains of tryptophan and tyrosine destabilized critical hydrogen bonds in the catalytic transition state. Consequently, these mutations reduced enzymatic activity, albeit improving substrate orientation. The structural predictions were corroborated by experimental enzymology metrics, cementing the role of aromatic residues in fine-tuning enzymatic product specificity, with geometric constraints acting as a double-edged regulatory sword—enhancing localization yet compromising catalytic competency.

### 3.4. Construction and Characterization of ReELP-Based Immobilization System

**Figure 2:**
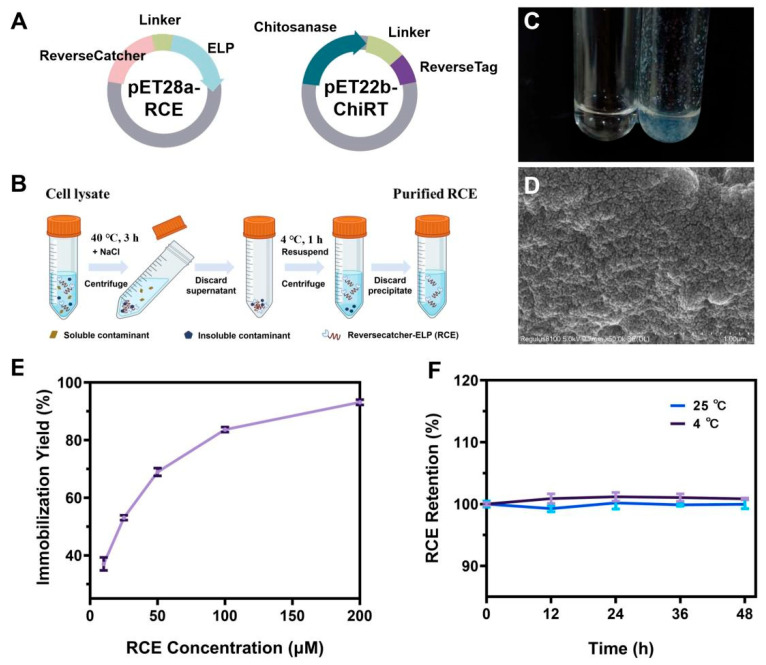
(**A**) Schematic diagram of RCE and ChitRT expression plasmids. (**B**) The specific mode of RCE purification. (**C**) Biosilica formed by RCE (left: normal PBS solution; right: RCE solution). (**D**) SEM micrograph of biosilica precipitated with RCE (scale bar 1 μm). (**E**) Immobilization efficiency at different RCE concentrations. (**F**) Leaching rates of proteins upon incubation in PBS (pH 6.0) at 4 °C and 25 °C.

For industrial enzyme catalysis, enzyme stability, reusability, and enzyme purification costs are crucial. Therefore, exploring a simple and universal technique for directly immobilizing target enzymes from cell lysates without the need for purification is a potential solution. Here, we utilized ReverseCatcher/ReverseTag and ELPs based system (ReELP system) to mediate one-step purification and immobilization CsnB-D78Y, the chitobiose synthase mutant with the highest activity in the aforementioned study. Specifically, combining CsnB-D78Y with a ReverseTag at its N-terminus (ChiRT), and coupling ReverseCatcher with ELPs to generate a silica-mineralizing carrier (RCE) (Figure 2A). The specific mode of RCE fusion protein purification was shown in Figure 2B. The RCE fusion protein demonstrated strong silicification capability, rapidly forming white silica precipitates under mild conditions, whereas no precipitation occurred in control reactions lacking RCE (Figure 2C). SEM analysis revealed that the resulting biosilica particles were spherical and possessed a rough surface, providing a large surface area favorable for enzyme immobilization (Figure 2D). Increasing RCE concentration promoted silica deposition and enhanced immobilization efficiency, reaching nearly 98% when 200 μM RCE was used (Figure 2E). Leakage tests confirmed the strong encapsulation of RCE within the silica matrix, as protein loss remained below 1% after 48 h of storage at either 4 °C or 25 °C (Figure 2F).

**Figure 3:**
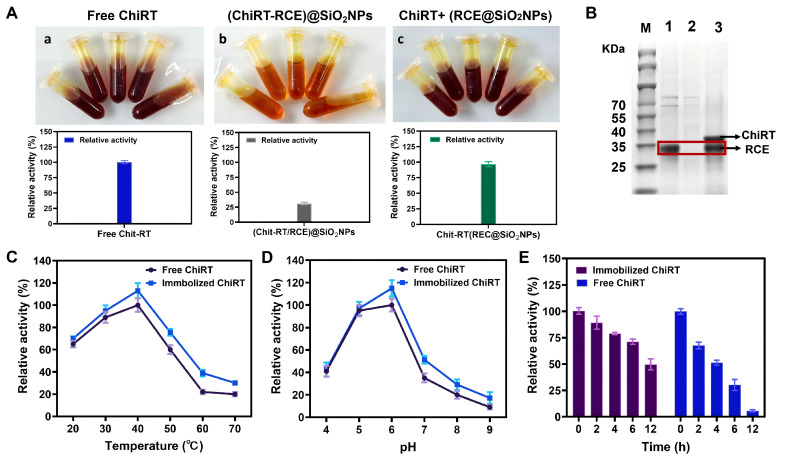
(**A**) Effect of immobilization modes on the enzyme activity of ChiRT ((**a**) free ChiRT enzyme; (**b**) ChiRT immobilized with RCE@SiO_2_ NPs; (**c**) ChiRT bound to RCE and then remineralized). (**B**) Analysis of purified Chit-RT immobilization by the SDS-PAGE. Red box revealed the position of the bands corresponding to ChitRT. Lane: M: molecular weight marker; 1: Purified Chit-RT; 2: the supernatant of purified ChitRT after immobilization employing RCE@SiO_2_; 3: the supernatant of ChiRT-RCE@SiO_2_ after dissolving with 100 mM NaOH. Effect of temperature (**C**), pH (**D**) and stability (**E**) of free and immobilized enzymes. Error bars: the standard deviation of triplicate assays.

To evaluate the influence of the enzyme loading and the sequence of ELP mineralization-immobilization on immobilization efficiency and enzyme activity, two immobilization strategies were compared: (1) coupling the enzyme to RCE prior to silica mineralization, and (2) mineralizing RCE first, followed by enzyme coupling. The first approach caused a severe ~70% loss of enzyme activity. We hypothesize that this reduction might be due to steric hindrance or active site blockage caused by the silica network forming around the enzyme during the mineralization process. In contrast, the second approach yielded optimal results with a relative activity of 96.8% (Figure 3A). We further used SDS-PAGE to verify whether ChitRT was successfully immobilized onto RCE. As shown in Figure 3B, after incubating RCE with ChiRT for 1 h and centrifuging, the band originally corresponding to the ChiRT protein almost disappeared in the supernatant. However, after dissolving ChiRT-RCE@SiO_2_ NPs with 100 mM NaOH, both the ChiRT protein band and the RCE protein band were detected. This indicates that RCE@SiO_2_ can directly and specifically bind to ChiRT through ReversCatcher/ReverseTag via covalent linkage.

Figure 3C illustrated the enzymatic activities of free and immobilized enzymes at various temperatures. The immobilized CsnB-D78Y (ChiRT-RCE@SiO_2_ NPs) demonstrated enhanced stability across a spectrum of environmental conditions in comparison to its free form. Temperature profile analysis revealed that both free and immobilized enzymes exhibited peak activity at 40 °C; nevertheless, immobilization resulted in improved relative activity at elevated temperatures, suggesting enhanced thermal resilience. These results indicate that the immobilized enzyme possesses superior temperature adaptability compared to the free enzyme, and that temperature variations do not compromise the conformational flexibility of the immobilized enzyme. The optimal pH for enzymatic activity primarily hinged on the properties of its functional groups, as well as the surface and residual charges of the immobilization matrix. Figure 3D depicted the activities of free and immobilized chitosanase at different pH levels. Compared to the free enzyme, the immobilized enzyme’s optimal pH remained unchanged, indicating that a mild immobilization method does not significantly shift the enzyme’s optimal pH. pH profile analysis demonstrated that immobilization enhanced pH tolerance, particularly in neutral and alkaline environments. This suggests that the silica carrier could prevent the denaturation of chitosanase under alkaline pH conditions. These findings imply that the silica matrix safeguards the enzyme against denaturation under extreme conditions.

Long-term stability tests further demonstrated the protective effect of RCE-based immobilization. After 12 h incubation at 40 °C and pH 6.0, the immobilized enzyme retained about 50% of its initial activity, while the free enzyme retained only 5.7% (Figure 3E). This improvement might be attributed to the silica carrier’s ability to stabilize the enzyme’s tertiary structure and shield it from unfolding.

### 3.5. ReELP-Based One Step Immobilization of CsnB-D78Y for Reusable Chitobiose Production

**Figure 4:**
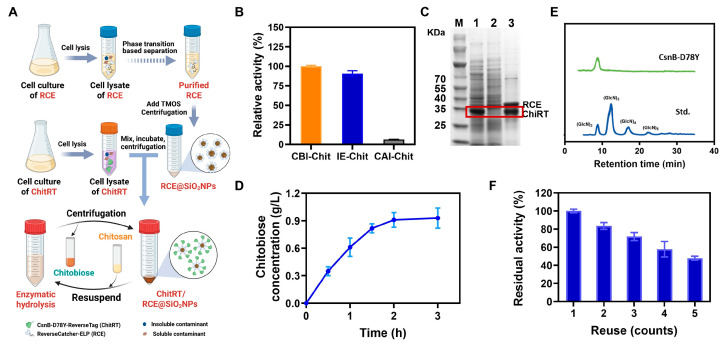
(**A**) Schematic illustration of ReELP-based one-step immobilization of ChiRT for reusable chitobiose production. (**B**) Enzymatic activity comparison of ChitRT before and after ReELP immobilization. CBI-Chit: Cell lysate supernatant before immobilization; IE-Chit: Immobilized enzymes; CAI-Chit: Cell lysate supernatant after immobilization. (**C**) Analysis of ChiRT cell lysate immobilization and purification by the SDS-PAGE. Red box revealed the position of the bands corresponding to ChitRT. Lane: M: molecular weight marker; 1: ChiRT cell lysate; 2: the supernatant of ChiRT cell lysate after immobilization employing RCE@SiO_2_; 3: the supernatant of ChiRT-RCE@SiO_2_ after dissolving with 100 mM NaOH. (**D**) Time curve of chitobiose production by ChiRT-RCE@SiO_2_. (**E**) HPLC analysis of products derived from hydrolysis of colloidal chitosan by ChiRT-RCE@SiO_2_. Std. represented standards. (**F**) Reusability of the immobilized CsnB-D78Y in PBS (pH 6.0) at 40 °C for 5 cycles. Error bars: the standard deviation of triplicate assays.

Given that ChiRT can specifically bind to RCE@SiO_2_ through the ReverseCatcher/ReverseTag system via covalent linkage, the evaluation was undertaken to assess the potentials of the ReELP system for directly employing ChiRT cell lysate to facilitate the purification, immobilization, and enable the cyclic catalytic synthesis of chitobiose from chitosan (Figure 4A). Initially, RCE@SiO_2_ was incubated with the supernatant of the cell lysate containing ChiRT for 1 h, followed by centrifugation to obtain the SiO_2_ NPs-immobilized enzyme. We measured and compared the enzymatic activities of the initial lysate supernatant, the immobilized enzyme, and the supernatant after centrifugation. As shown in Figure 4B, the immobilization efficiency of the enzyme in the lysate supernatant reached 96.8%, with an enzyme activity recovery rate of 90.7%. The immobilization and purification results were further verified by SDS-PAGE. As shown in Figure 4C, the supernatant of ChiRT cell lysate after employing RCE@SiO_2_ revealed the loss of Chit-RT band (Lane 1 and 2). After dissolving the nanoparticles with 100 mM NaOH, both the ChiRT protein band and the RCE protein band were detected (Lane 3). Accordingly, it clearly revealed that RCE can specifically and covalently react with the ChiRT (via ReverseCatcher and ReverseTag) from the cell lysate in one-step.

Furthermore, the immobilized enzymes were employed to catalyze the synthesis of chitobiose from a 1 g/L chitosan substrate. As illustrated in Figure 4D, the chitobiose yield approached its peak after 2 h, reaching 0.91 g/L with a conversion of 91.0%. HPLC analysis (Figure 4E) revealed that the reaction product was nearly exclusively chitobiose, with a purity exceeding 98%. These results further confirm the high efficiency and specificity of CsnB-D78Y in chitobiose synthesis. Reusability assays confirmed the operational advantages of the immobilized enzyme. Over 5 consecutive hydrolysis cycles, the immobilized enzyme consistently converted chitosan into chitobiose with high efficiency, retaining approximately 50% of its initial activity (Figure 4F). The observed decline in activity might be attributed to the following factors: (1) the relatively low thermostability of CsnB-D78Y; (2) loss of nanoparticles during centrifugation and washing processes [34,35]; (3) potential obstruction of ChiRT active sites and conformational changes caused by the co-immobilization of RCE and ChiRT [36]; and (3) slight leakage of RCE@SiO_2_ in the buffer solution, which may also contribute to the loss of activity. To further enhance the feasibility of the industrial application of this chitobiose production system, future work should focus on the modification of CsnB-D78Y to improve its inherent thermal stability.

## 4. Conclusions

This study successfully integrated rational protein engineering with an innovative ReELP-based immobilization system to achieve specific and sustainable chitobiose production from chitosan. Through structure-guided design targeting substrate-binding subsites, we developed the CsnB-D78Y mutant that exclusively produces chitobiose with superior catalytic performance (102.4 U/mg), surpassing previously reported chitobiose-specific enzymes. The ReELP system integrated elastin-like polypeptides with the ReverseCatcher/ReverseTag peptide pair, enabling direct purification, immobilization and cyclic synthesis of chitobiose from crude cell lysate. It achieved high immobilization efficiency (96.8%) and activity (>90.7%), with enhanced enzyme stability. Immobilized CsnB-D78Y showed improved thermal and pH tolerance, retaining 50% activity after 12 h at 40 °C vs. 5.7% for free enzymes. Using this system, 91% chitobiose conversion and >98% purity were achieved, demonstrating a robust platform for industrial-scale production.

This integrated approach can be extended to other glycoside hydrolases for producing COSs with defined DPs, expanding the toolkit for controlled chitosan depolymerization. Future work will focus on enhancing enzyme thermostability and optimizing immobilization matrices to further improve long-term operational stability for industrial implementation.

## Data Availability

The original contributions presented in this study are included in the article/Supplementary Material. Further inquiries can be directed to the corresponding author.

## Supplementary Materials

The following supporting information can be downloaded at: https://www.mdpi.com/article/10.3390/foods14244248/s1, Table S1: Primers used for plasmids construction; Figure S1: The hydrolytic products analysis of the CsnB and its mutants by thin layer chromatography.

## Abbreviations

The following abbreviations are used in this manuscript:

COSs	Chitosan oligosaccharides
DP	Degrees of polymerization
GlcNAc	N-acetyl glucosamine
GlcN	Glucosamine
ELP	Elastin-like polypeptide
ITT	Inverse temperature transition
(GlcN)_2_	Chitobiose
(GlcN)_3_	Chitotriose
(GlcN)_4_	Chitotetraose
(GlcN)_5_	Chitopentaose
(GlcN)_6_	Chitohexaose
RCE	ReverseCatcher-ELP
ChiRT	CsnB-D78Y-ReverseTag
DNS	3,5-dinitrosalicylic acid
NPs	Nanoparticles
TLC	Thin layer chromatography
ReELP system	ReverseCatcher/ReverseTag and ELPs based system
HPLC	High performance liquid chromatography
