# Supplementary material for: Rational Design and One-Step Immobilization of Chitosanase for Specific and Recyclable Chitobiose Production
Source: Foods. 2025 Dec 10;14(24):4248. doi: 10.3390/foods14244248 (PMC12732231; doi:10.3390/foods14244248)
Supplement: Supplementary file 1 [file foods-14-04248-s001.zip › foods-4022376-supplementary.pdf]

Supplementary Materials

Rational Design and One-Step Immobilization of Chitosanase for Specific and Recyclable Chitobiose Production

Dandan Tang[1,2], Jie Zhang[1], Na Li[1], Rui Long[1], Xinyu Wang[1], Xiaowen Wang[1], Wei Liu[1,*]

[1] College of Food Science and Light Industry, Nanjing Tech University, Nanjing 211816, China;

[2] School of Pharmaceutical Sciences, Nanjing Tech University, Nanjing 211816, China

[*] Corresponding author: Wei Liu, E-mail: liuwei6775@njtech.edu.cn.

Table S1 Primers used for plasmids construction

Plasmids	Primers	Sequences (5’→3’)
pET-22b-CsnB-D78Y	D-F-Y	CCGGAACAGTATGATCTGAATTGGATTAAATATTACGG
	D-R-Y	TCAGATCATACTGTTCCGGCTTGTTGATC
pET-22b-CsnB-D78W	D-F-W	CCGGAACAGTGGGATCTGAATTGGATTAAATATTACGG
	D-R-W	ATTCAGATCCCACTGTTCCGGCTTGTTGATC
pET-22b-CsnB-K260W	K-F-W	AAATATAATTGGCCGCCGAATGGCAAAAATC
	K-R-W	TTCGGCGGCCAATTATATTTATTGGTATCAACAACCAGG
pET-22b-CsnB-K260Y	K-F-Y	AAATATAATTATCCGCCGAATGGCAAAAATC
	K-R-Y	ATTCGGCGGATAATTATATTTATTGGTATCAACAACCAGG
pET-22b-CsnB-P115A	P-F-A	GATACCCATGCTGATGGCCCGGATCTGTTC
	P-R-A	CGGGCCATCAGCATGGGTATCGCGACTGC
pET-22b-CsnB-H114W	H-F-W	CGCGATACCTGGCCGGATGGCCCGG
	H-R-W	GCCATCCGGCCAGGTATCGCGACTGCCG
pET-22b-CsnB-A218D	A-F-D	ATCAGGGCGATACCGGTGGCAGCG
	A-R-D	GCCACCGGTATCGCCCTGATTCAGGGC
pET-22b-CsnB-A218K	A-F-K	AATCAGGGCAAGACCGGTGGCAGCG
	A-R-K	GCCACCGGTCTTGCCCTGATTCAGGGCG
pET-22b-CsnB-A218R	A-F-R	AATCAGGGCCGTACCGGTGGCAGCG
	A-R-R	GCCACCGGTACGGCCCTGATTCAGGGCG
pET-22b-CsnB-A218S	A-F-S	AATCAGGGCAGCACCGGTGGCAGCG
	A-R-S	CCACCGGTGCTGCCCTGATTCAGGGCG
pET-22b-CsnB-A218T	A-F-T	AATCAGGGCACCACCGGTGGCAGC
	A-R-T	CACCGGTGGTGCCCTGATTCAGGGCG
pET-22b-CsnB-G220Y	G-F-Y	GGCGCCACCTATGGCAGCGATACCCTG
	G-R-Y	TCGCTGCCATAGGTGGCGCCCTGATTC
pET-22b-CsnB-T219Y	T-F-Y	CAGGGCGCCTACGGTGGCAGCGATACC
	T-R-Y	CTGCCACCGTAGGCGCCCTGATTCAGG
pET-22b-CsnB-G220W	G-F-W	GGCGCCACCTGGGGCAGCGATACCCTG
	G-R-W	TCGCTGCCCCAGGTGGCGCCCTGATTCA
pET-22b-CsnB-T219W	T-F-W	CAGGGCGCCTGGGGTGGCAGCGATACC
	T-R-W	GCTGCCACCACCGGCGCCCTGATTCAGGG
pET-28a-Reverse Catcher-ELP	RC-RCE-F	CTTTAAGAAGGAGATATACATATGACCATTCCGGAAGTTAAAGAAG
	RC-RCE-R	GAACCTGATCCTGACCCGGATCCTTCGTAGTTATTATCGGTATC
	Gj-RCE-F	GGATCCGGGTCAGGATCAGGTTCAGGCTCATCTAAAGGTCCGGGTGT
	Gj-RCE-R	CATATGTATATCTCCTTCTTAAAG
pET-22b-Reverse Tag-CsnB-D78Y	ChiRT-F	CAGGTGGTGAAACATGAAGATAAAAACGATAAAGCGCAGACCCTGATTGTGGAAAAACCGAATCGCTGAGATCCGGCTGCTAACAAAGCC

	ATCTTCATGTTTCACCACCTGTTTGGTATCACTGCCAC
ChiRT-R	CGCCACCGCTACCGCCACCGCCATCCCACTGTTTAAC ACGAT

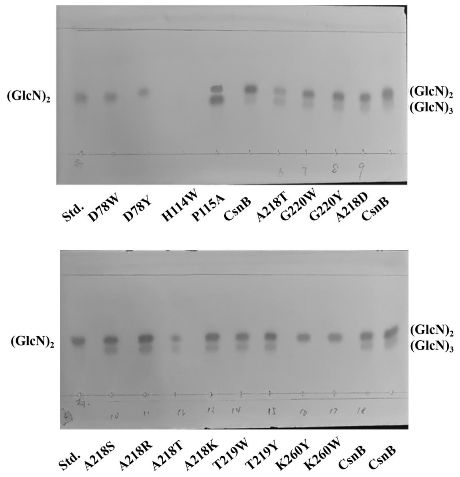

Figure S1 The hydrolytic products analysis of the CsnB and its mutants by thin layer chromatography
